# Metastatic Parathyroid Carcinoma in a Young Adult With Recurrent Hypercalcemia and Acute Necrotizing Pancreatitis

**DOI:** 10.7759/cureus.79942

**Published:** 2025-03-03

**Authors:** Moemen Hasaballah, Mohamed Abdulmajeed, Amira Abohegazy

**Affiliations:** 1 General Internal Medicine, Luton and Dunstable University Hospital, Luton, GBR; 2 Pharmacology, Faculty of Medicine, Menofia University, Al Minufiyah, EGY

**Keywords:** acute necrotizing pancreatitis, adult primary hyperparathyroidism, hypercalcemia, lung metastasis, parathyroid carcinoma

## Abstract

Parathyroid carcinoma (PC) is an extremely rare form of endocrine cancer, accounting for a very small proportion of primary hyperparathyroidism cases. Even more extraordinary is metastatic PC presenting with complications such as severe hypercalcemia and pancreatitis. In this report, we present the case of a 33-year-old male patient who developed metastatic PC with recurrent hypercalcemia and acute necrotizing pancreatitis. This case sheds light on the complexities of diagnosing and managing this rare and aggressive condition.

## Introduction

Parathyroid carcinoma (PC) is a very rare malignancy, accounting for fewer than 1% of all cases of primary hyperparathyroidism worldwide [1,2]. The disease presents a diagnostic and therapeutic challenge, often mimicking more common benign conditions such as parathyroid adenomas. While localized PC is already difficult to manage, metastatic PC is an even rarer and more aggressive form, associated with severe complications such as life-threatening hypercalcemia, renal failure, and acute pancreatitis [2,3].

In clinical practice, hypercalcemia is a hallmark of parathyroid malignancy and, when severe, can have profound systemic effects. One of its most dangerous yet uncommon manifestations is acute pancreatitis, which can progress to necrotizing pancreatitis, as seen in our patient. The pathophysiology of hypercalcemia-induced pancreatitis is not well understood, but calcium is believed to play a crucial role in pancreatic enzyme activation and tissue destruction [3].

This case underscores the unpredictable and severe nature of metastatic PC, particularly in young patients, where delayed diagnosis or inadequate follow-up can lead to devastating consequences. Our report details the clinical course of a 33-year-old male who presented with recurrent hypercalcemia along with acute necrotizing pancreatitis and metastatic spread of PC. Through this case, we highlight the critical importance of early diagnosis, vigilant long-term follow-up, and emerging therapeutic strategies for this rare but life-threatening condition.

## Case presentation

A 33-year-old male came to the emergency department with severe abdominal pain radiating to his back, along with an episode of fainting. His medical history was significant and complex. In 2014, he was diagnosed with a parathyroid adenoma after experiencing acute pancreatitis and acute kidney injury (AKI) caused by hypercalcemia. He underwent a right inferior parathyroidectomy and was started on cinacalcet and amlodipine. In 2017, his condition progressed to PC, requiring a right hemithyroidectomy and radiotherapy. After moving to the United Kingdom in 2022, he did not attend regular blood tests or follow-up appointments.

The patient was admitted to our hospital in November 2024. On examination, the patient was unable to lie flat due to intense abdominal and back pain and looked severely dehydrated. His vital signs were stable, with a blood pressure of 117/72 mmHg, a heart rate of 70 beats per minute, oxygen saturation at 97%, and a temperature of 36.5°C. Chest examination showed normal air entry with normal chest expansion and no adventitious sounds. Abdominal examination showed very severe tenderness in the epigastric area.

Laboratory tests showed extremely high calcium levels (4.45 mmol/L), elevated parathyroid hormone (PTH), and markers of acute pancreatitis and kidney dysfunction. These findings are summarized in Table 1.

**Table 1:** Laboratory test results.

Test	Result	Normal range
Calcium	4.45 mmol/L	2.2–2.6 mmol/L
Phosphate	0.73 mmol/L	0.8–1.5 mmol/L
Magnesium	0.55 mmol/L	0.7–1 mmol/L
Lipase	1,586 U/L	0–60 U/L
Parathyroid hormone	13.9 pmol/L	1.6–6.9 pmol/L
Creatinine	265 µmol/L	62–106 µmol/L
Urea	9.3 mmol/L	2.5–7.8 mmol/L
Vitamin D	92 nmol/L	50–200 nmol/L
White blood cell count	13.3 × 10^9^/L	4–11 × 10^9^/L
Hemoglobin	136 g/L	130–165 g/L
Platelets	204 × 10^9^/L	150–450 × 10^9^/L
C-reactive protein	445 mg/L	0–4.9 mg/L
Glucose (random plasma)	7.2 mmol/L	Below 11.1 mmol/L
International normalized ratio	1.1	0.8–1.2
Alkaline phosphatase	180 U/L	30–130 U/L
Alanine aminotransferase	18 U/L	0–40 U/L

**Figure 1:**
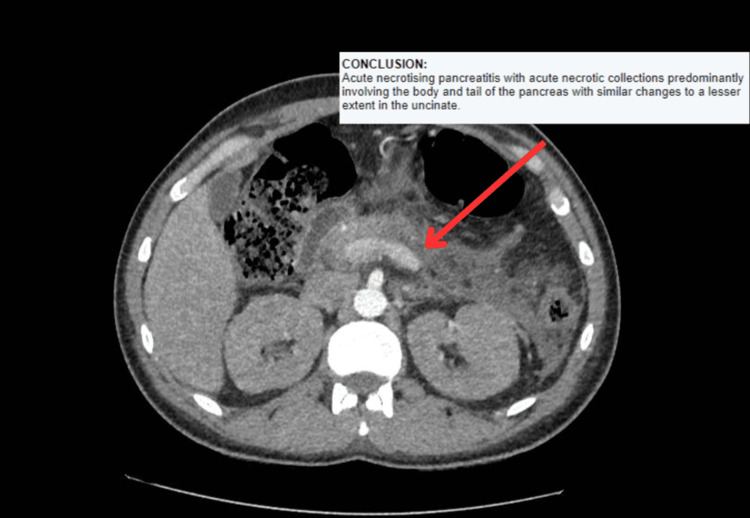
CT of the abdomen showing acute necrotizing pancreatitis.

**Figure 2:**
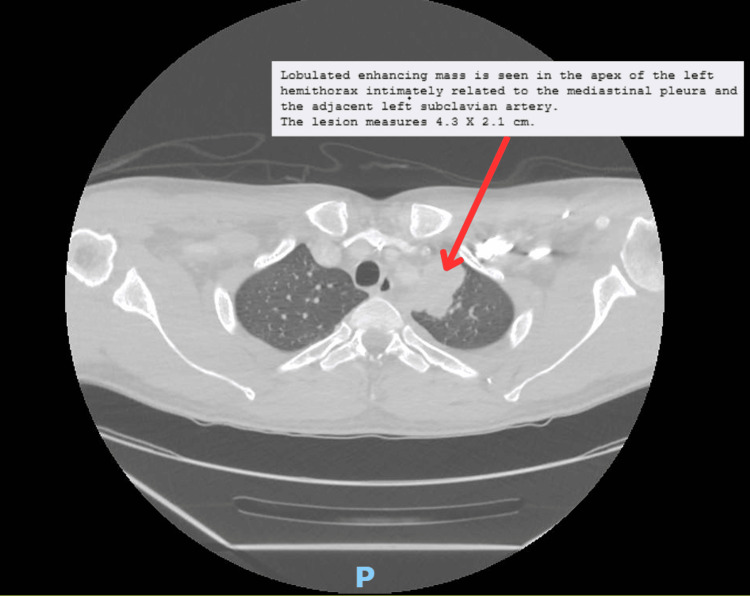
CT of the chest showing a left lung mass.

Further investigations were done including CT of the abdomen which showed necrotizing pancreatitis with peripancreatic fluid collections (Figure 1). The CT of the abdomen also ruled out any obstructive diseases, such as stones or cancers. Additionally, a CT of the chest revealed a lobulated mass in the left hemithorax, measuring 4.3 × 2.1 cm (Figure 2).

A respiratory and endocrine multidisciplinary team meeting was held to discuss the patient’s diagnosis, and the decision was made to take a CT-guided biopsy of the left upper lobe. The histopathology results indicated the presence of malignant epithelioid neoplasm with solid and nested growth patterns in the lung parenchyma morphologically consistent with metastatic PC. No tumor necrosis or lymphvascular invasion was seen.

## Discussion

PC is extremely rare, and even among those diagnosed, metastasis is infrequent. This patient’s case stands out because his metastatic PC manifested in such an unusual and severe way [3,4].

Hypercalcemia and pancreatitis

Severe hypercalcemia, as seen in this patient, is the hallmark of PC. When calcium levels spike to such dangerous levels, they can lead to acute complications, including pancreatitis. Although hypercalcemia-induced pancreatitis is rare, it is believed to occur due to calcium’s activation of pancreatic enzymes, leading to tissue damage [4,5].

Diagnostic and management challenges

Diagnosing metastatic PC presents significant challenges, particularly when it involves metastasis to the lungs. The condition can often mimic more benign disorders, and the metastatic lesions may be difficult to identify on imaging studies [6]. In this case, the thoracic mass required histopathological confirmation to definitively diagnose the presence of metastasis. Identifying such metastatic lesions in the lung can be particularly challenging, as they may mimic other more common pulmonary conditions, and may not always be easily distinguishable without tissue biopsy.

**Figure 3:**
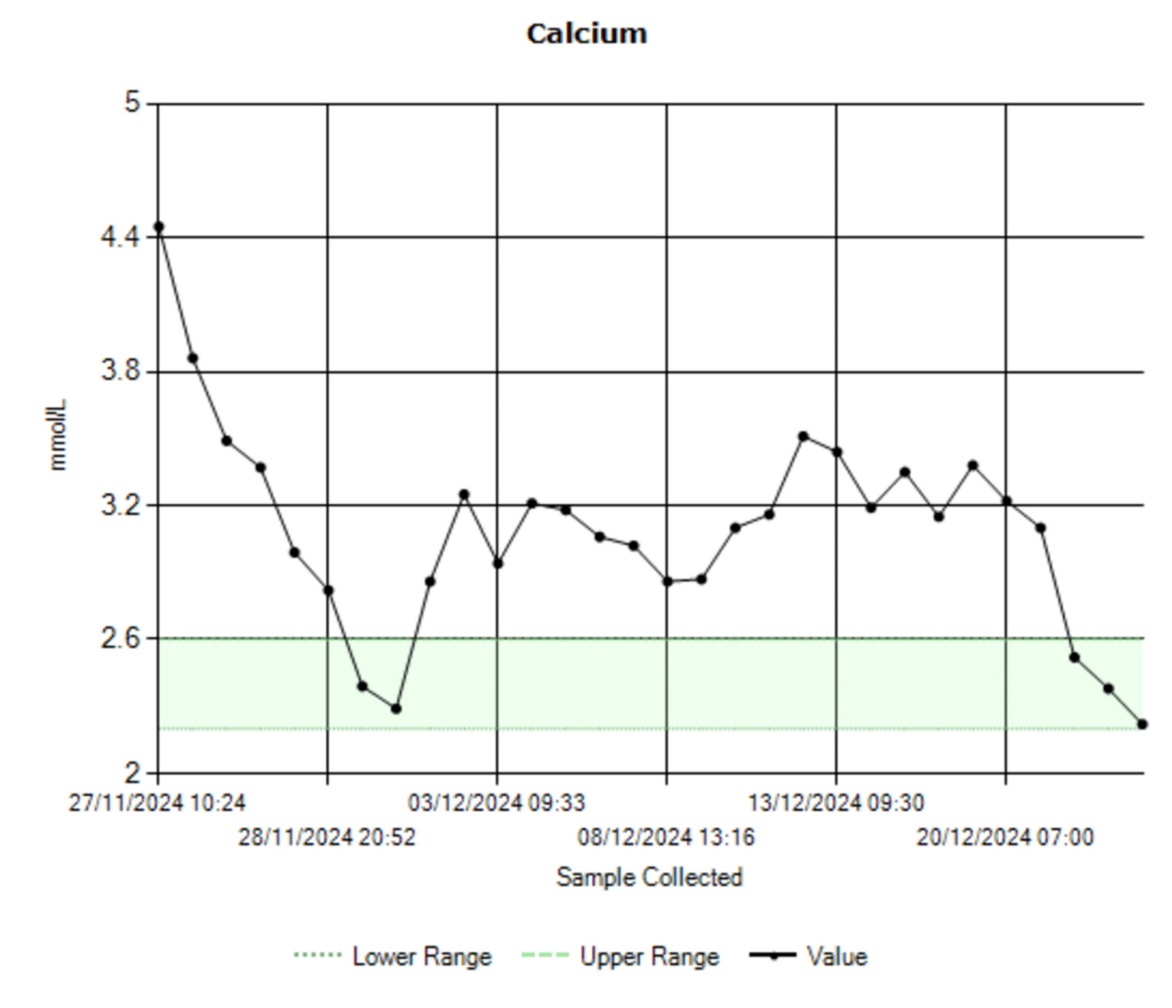
Calcium trend.

The patient’s management focused on controlling the patient’s hypercalcemia and slowing the progression of the disease, when possible. To address the hypercalcemia, the patient received aggressive hydration with intravenous fluids and bisphosphonates (pamidronate and zoledronate) to lower the calcium levels, which dropped from 4.45 mmol/L to 2.22 mmol/L (Figure 3). Intravenous phosphate and magnesium were also given to correct any deficiencies.

**Figure 4:**
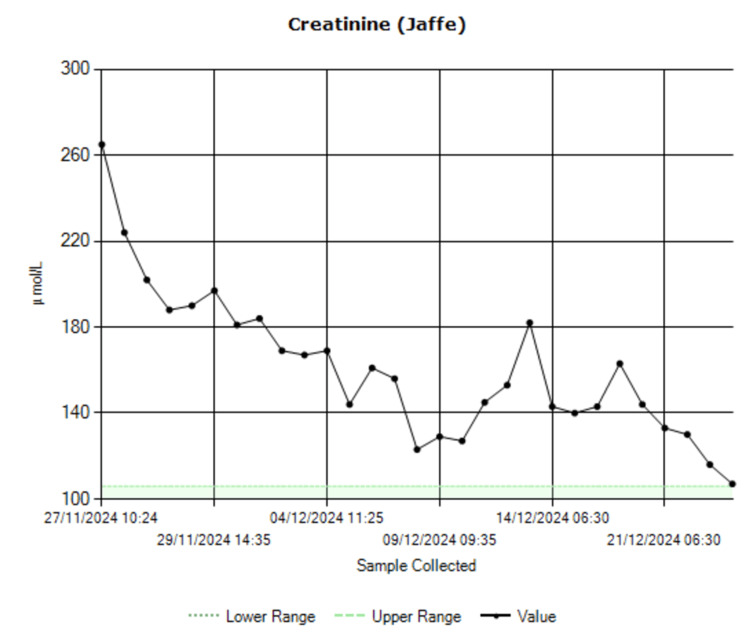
Creatinine trend.

In light of the patient’s acute pancreatitis, it was treated with a seven-day course of piperacillin-tazobactam 4.5g three times daily. The AKI resolved, with creatinine levels improving from 265 µmol/L at admission to 107 µmol/L at discharge (Figure 4).

For ongoing care, the patient continued cinacalcet to help reduce PTH levels, along with amlodipine 5 mg once daily. Because of the extent of the metastatic disease, curative surgery was not an option. However, the team held a multidisciplinary meeting to discuss the possibility of surgical intervention and agreed on debulking surgery after counselling the patient about the possible treatment options [3,5].

Prognosis and emerging therapies

Metastatic PC generally carries a poor prognosis, with a 10-year survival rate of only around 50% [2]. While current treatment options primarily involve a combination of medication and surgery, these approaches often offer limited success, especially in advanced stages of the disease. However, there is hope on the horizon with emerging therapies. Early clinical trials are showing promise for new treatments that target the calcium-sensing receptor and PTH-related pathways, which play a key role in the development and progression of the disease. Some of these emerging therapies include calcimimetics such as cinacalcet, which help regulate PTH production and manage hypercalcemia, and PTHrP inhibitors, aimed at blocking the peptide involved in tumor growth and metastasis. Additionally, targeted therapies, such as tyrosine kinase Inhibitors (e.g., sunitinib) and immune checkpoint inhibitors (e.g., pembrolizumab), are being explored for their ability to slow tumor progression and enhance immune responses. These innovative therapies could potentially improve outcomes by more effectively controlling the underlying mechanisms driving tumor growth and metastasis. While still in the early stages, these advances offer hope for better management of metastatic PC in the future [6].

## Conclusions

Metastatic PC is an exceptionally rare and aggressive malignancy that presents significant diagnostic and management challenges. This case highlights the severe complications of recurrent hypercalcemia, including acute necrotizing pancreatitis, in a young patient with metastatic PC. Hypercalcemia, a hallmark of the disease, can lead to life-threatening systemic effects, making timely diagnosis and intervention critical. Unfortunately, due to the patient’s lack of regular follow-up, his condition progressed unnoticed, emphasizing the need for continuous biochemical monitoring in patients with a history of PC.

Despite advances in endocrine oncology, the prognosis for metastatic PC remains poor, with limited curative options. While surgical intervention remains the gold standard for localized disease, metastatic cases require a multimodal approach, including aggressive management of hypercalcemia and emerging targeted therapies. This case highlights the importance of early detection, vigilant long-term follow-up, and evolving treatment strategies to improve patient outcomes. Continued research into novel therapeutic approaches offers hope for better disease control and survival in this challenging malignancy.
